# Complete genome sequences of 30 bacterial species from a synthetic community

**DOI:** 10.1128/mra.00111-24

**Published:** 2024-05-10

**Authors:** Shane L. Hogle, Manu Tamminen, Teppo Hiltunen

**Affiliations:** 1 Department of Biology, University of Turku, Turku, Finland; California State University San Marcos, San Marcos, California, USA

**Keywords:** bacterial genome assembly, synthetic communities, experimental evolution

## Abstract

We present complete genome sequences from 30 bacterial species that can be used to construct defined synthetic communities that stably form in the laboratory under controlled conditions.

## ANNOUNCEMENT

Synthetic bacterial communities are defined collections of bacteria with desirable properties for laboratory experimentation. They allow for the controlled manipulation of species and their environment, which can provide insight into the governing principles of a system (1, 2). DNA sequencing is a commonly used tool to characterize synthetic bacterial communities. For example, 16S rRNA amplicon sequencing can provide information about community composition, while whole-genome sequencing (WGS) can reveal the genetic changes underlying microbial adaptation. However, WGS experiments require high-quality reference genomes to reliably identify and annotate genetic mutations. To this end, we report the closed genomes from 30 bacterial species (Table 1) that can be readily used to construct synthetic bacterial communities.

**TABLE 1:** Summary data for the 30 HAMBI species

Species name	HAMBI ID/strain	GenBank accession no.	SRA accession no. (short read, long read)	Illumina sequencing technology	Illumina read pairs	Illumina coverage (×)	Long-read sequencing technology^*a*^	PacBio/ONT reads	PacBio/ONT mean length (bp)	PacBio/ONT coverage (×)	Assembly N50 (bp)	Assembly size (bp)	Total contigs	Chromosomal contigs^*b*^	Plasmid contigs	GC content (%)	Genes	CDS^*c*^	16S rRNA genes	CheckM completeness	GTDB v207.2 species assignment	GTDB terminal branch ANI^*d*^
*Acinetobacter* *johnsonii*	HAMBI_0097	GCA_034424475.1	SRR27033697, SRR27033696	MiSeq, 2 × 300 bp	3,698,543	633	PacBio HiFi	60,377	10,854	187	3,369,756	3,507,189	3	1	2	41.5	3474	3,146	7	99.73	*Acinetobacter johnsonii*	95.98
*Aeromonas* *caviae*	HAMBI_1972	GCA_034424415.1	SRR27033671, SRR27033670	MiSeq, 2 × 300 bp	637,848	84	PacBio HiFi	76,038	9,553	159	4,578,158	4,578,158	1	1	0	61.5	4240	4,038	10	99.97	*Aeromonas caviae*	99.99
*Agrobacterium* *tumefaciens*	HAMBI_0105	GCA_034424685.1	SRR27033723, SRR27033721	MiSeq, 2 × 300 bp	690,772	76	PacBio HiFi	60,204	11,823	131	2,954,609	5,423,958	3	2	1	59.5	5163	5,029	4	*100*	*Agrobacterium tumefaciens*	98.02
*Bordetella avium*	HAMBI_2160	GCA_034424645.1	SRR27033718, SRR27033717	MiSeq, 2 × 300 bp	712,180	115	PacBio HiFi	63,167	11,318	192	3,721,798	3,721,798	1	1	0	61.5	3446	3,333	3	100	*Bordetella avium*	100
*Brevundimonas* *bullata*	HAMBI_0262	GCA_034424665.1	SRR27033693, SRR27033692	MiSeq, 2 × 300 bp	3,165,389	545	PacBio HiFi	52,730	12,140	184	3,484,100	3,484,100	1	1	0	67	3373	3,267	3	*99.68*	*Brevundimonas bullata*	100
*Brevundimonas* *diminuta*	HAMBI_0018	GCA_034424705.1	SRR27033719, SRR27033708	Nextseq 2000, 2 × 150 bp	2,260,337	198	ONT	156,497	5,867	268	3,423,586	3,423,586	1	1	0	67.5	3415	3,335	2	100	*Brevundimonas diminuta*	99.79
*Chitinophaga* *sancti*	HAMBI_1988	GCA_034424315.1	SRR27033667, SRR27033666	MiSeq, 2 × 300 bp	692,695	49	PacBio HiFi	80,087	11,068	105	8,421,768	8,421,768	1	1	0	44	6638	6,542	5	99.84	*Chitinophaga sancti*	99.99
*Citrobacter koseri*	HAMBI_1287	GCA_034424455.1	SRR27033687, SRR27033686	MiSeq, 2 × 300 bp	673,789	85	PacBio HiFi	69,401	12,212	179	4,579,071	4,732,180	3	1	2	54	4483	4,323	7	99.88	*Citrobacter_B koseri*	98.8
*Comamonas testosteroni*	HAMBI_0403	GCA_034424235.1	SRR27033691, SRR27033690	MiSeq, 2 × 300 bp	588,427	60	PacBio HiFi	69,878	11,506	137	5,812,347	5,852,547	2	1	1	61.5	5388	5,139	9	99.85	*Comamonas testosteroni_C*	95.12
*Cupriavidus oxalaticus*	HAMBI_2164	GCA_034424395.1	SRR27033716, SRR27033715	MiSeq, 2 × 300 bp	734,486	66	PacBio HiFi	70,249	10,726	113	3,884,110	6,693,411	2	2	0	67	6086	5,925	5	99.94	*Cupriavidus oxalaticus*	100
*Escherichia coli*	ATCC 11303	GCA_034424725.1	SRR27033725, SRR27033724	Nextseq 2000, 2 × 150 bp	8,256,097	536	ONT	91,572	5,075	101	4,619,496	4,619,494	1	1	0	51	4485	4,193	7	100	*Escherichia coli*	96.82
*Hafnia alvei*	HAMBI_1279	GCA_034424155.1	SRR27033689, SRR27033688	MiSeq, 2 × 300 bp	669,969	82	PacBio HiFi	93,416	10,477	200	4,728,715	4,884,924	2	1	1	49	4502	4,320	7	99.92	*Hafnia alvei*	100
*Janthinobacterium* *lividum*	HAMBI_1919	GCA_034424625.1	SRR27033676, SRR27033675	Nextseq 2000, 2 × 150 bp	2,909,325	129	ONT	152,719	5,732	129	6,322,531	6,762,807	2	1	1	62.5	6014	5,840	8	99.65	*Janthinobacterium lividum*	100
*Kluyvera* *intermedia*	HAMBI_1299	GCA_034424175.1	SRR27033682, SRR27033681	MiSeq, 2 × 300 bp	559,655	71	PacBio HiFi	70,571	10,993	164	4,732,808	4,738,233	2	1	1	52.5	4545	4,306	8	99.4	*Kluyvera intermedia*	99.97
*Microvirga lotononidis*	HAMBI_3237	GCA_034627025.1	SRR27033703, SRR27033702	MiSeq, 2 × 300 bp	560,102	46	PacBio HiFi	76,827	10,886	114	4,965,092	7,316,924	4	3	1	63	7090	6,706	6	99.69	*Microvirga lotononidis*	99.94
*Moraxella canis*	HAMBI_2792	GCA_034424565.1	SRR27033707, SRR27033706	MiSeq, 2 × 300 bp	497,250	136	PacBio HiFi	88,826	10,252	416	2,178,558	2,187,333	3	1	2	45	1985	1,899	4	99.73	*Moraxella canis*	97.69
*Morganella morganii*	HAMBI_1292	GCA_034424335.1	SRR27033684, SRR27033683	MiSeq, 2 × 300 bp	594,696	94	PacBio HiFi	85,629	10,436	234	3,814,514	3,814,514	1	1	0	51	3648	3,507	7	100	*Morganella morganii*	99.99
*Myroides odoratus*	HAMBI_1923	GCA_034424295.1	SRR27033673, SRR27033672	Nextseq 2000, 2 × 150 bp	2,407,984	170	ONT	195,631	5,786	267	4,243,755	4,243,755	1	1	0	36	3798	3,661	6	99.65	*Flavobacterium odoratum*	99.97
*Niabella* *yanshanensis*	HAMBI_3031	GCA_034424215.1	SRR27033705, SRR27033704	MiSeq, 2 × 300 bp	596,448	65	PacBio HiFi	86,032	11,164	173	5,543,186	5,543,186	1	1	0	42.5	4577	4,515	2	100	*Niabella yanshanensis*	100
*Novosphingobium* *capsulatum*	HAMBI_0103	GCA_034424435.1	SRR27033695, SRR27033694	Nextseq 2000, 2 × 150 bp	2,823,704	171	ONT	133,813	6,165	166	4,093,864	4,961,597	3	1	2	65.6	4528	4,409	4	99.42	*Novosphingobium capsulatum*	100
*Paraburkholderia* *kururiensis*	HAMBI_2494	GCA_034424375.1	SRR27033712, SRR27033711	MiSeq, 2 × 300 bp	421,884	38	PacBio HiFi	61,696	11,935	110	6,677,280	6,711,709	2	1	1	65.5	5987	5,839	4	99.95	*Paraburkholderia kururiensis_A*	100
*Paracoccus* *denitrificans*	HAMBI_2443	GCA_034627565.1	SRR27033714, SRR27033713	MiSeq, 2 × 300 bp	770,622	88	PacBio HiFi	77,753	9,973	148	2,853,118	5,237,024	3	2	1	67	5219	5,042	3	99.7	*Paracoccus* *denitrificans*	100
*Pseudomonas* *chlororaphis*	HAMBI_1977	GCA_034424585.1	SRR27033669, SRR27033668	MiSeq, 2 × 300 bp	780,085	70	PacBio HiFi	62,998	11,555	109	6,670,897	6,670,897	1	1	0	63	6035	5,884	5	100	*Pseudomonas_E chlororaphis*	97.26
*Pseudomonas* *marginalis*	SBW25	GCA_034424355.1	SRR27033701, SRR27033700	Nextseq 2000, 2 × 150 bp	3,476,023	155	ONT	100,443	6,530	98	6,722,400	6,722,400	1	1	0	60.5	6165	5,984	5	99.93	*Pseudomonas_E marginalis_B*	98.28
*Pseudomonas* *putida*	HAMBI_0006	GCA_034424745.1	SRR27033685, SRR27033674	Illumina MiSeq, 2 × 300 bp	531,769	49	PacBio HiFi	60,163	11,913	109	6,559,726	6,559,808	1	1	0	61.5	6082	5,864	7	99.88	*Pseudomonas_E putida*	98.48
*Serratia marcescens* subsp. *marcescens*	ATCC 13880	GCA_034424255.1	SRR27033699, SRR27033698	Nextseq 2000, 2 × 150 bp	3,755,783	218	ONT	135,635	5,798	152	5,117,320	5,160,509	2	1	1	60	4933	4,775	7	99.8	*Serratia marcescens*	100
*Sphingobacterium spiritivorum*	HAMBI_1896	GCA_034424195.1	SRR27033678, SRR27033677	MiSeq, 2 × 300 bp	656,566	77	PacBio HiFi	85,440	10,252	171	5,136,875	5,136,875	1	1	0	40	4355	4,275	4	99.84	*Sphingobacterium spiritivorum*	99.99
*Sphingobium yanoikuyae*	HAMBI_1842	GCA_034424525.1	SRR27033680, SRR27033679	MiSeq, 2 × 300 bp	880,164	96	PacBio HiFi	63,512	10,496	121	5,155,848	5,527,200	7	1	6	64.5	5212	5,071	4	99.59	*Sphingobium yanoikuyae*	99.99
*Stenotrophomonas maltophilia*	HAMBI_2659	GCA_034424605.1	SRR27033710, SRR27033709	MiSeq, 2 × 300 bp	692,990	83	PacBio HiFi	57,800	11,510	133	5,004,263	5,004,264	1	1	0	66	4671	4,521	4	100	*Stenotrophomonas maltophilia*	100
*Trinickia* *caryophylli*	HAMBI_2159	GCA_034424545.1	SRR27033722, SRR27033720	MiSeq, 2 × 300 bp	750,507	68	PacBio HiFi	102,397	8,323	129	4,294,907	6,589,286	2	2	0	64.5	5839	5,684	4	99.47	*Trinickia caryophylli*	100

^*a*^ ONT, Oxford Nanopore Technologies.

^*b*^ Chromosomal contigs are operationally defined as having length > 1 Mbp and with chromosomal assignment from MOB Recon.

^*c*^ CDS, coding sequence.

^*d*^ Average nucleotide identity between query and reference genome at a terminal branch of the GTDB taxonomy.

Bacterial species were selected from the University of Helsinki Microbial Domain Biological Resource Centre (HAMBI) because they could grow in the same shared medium under standard laboratory conditions, most could coexist in liquid culture over extended periods (3), and all could be uniquely distinguished by 16S rRNA amplicon sequencing (4). Each species was sequenced using one short-read (either Illumina MiSeq or NextSeq 2000) and one long-read (Oxford Nanopore MinION or PacBio Sequel II) approach (Table 1). Bacteria were grown in proteose peptone yeast extract medium for 24–48 hours at 30°C, and DNA was extracted using the ZymoBIOMICS DNA Miniprep Kit (for PacBio, MinION, and NextSeq) or the Qiagen DNeasy Blood & Tissue Kit (MiSeq). MiSeq libraries used the Nextera XT DNA Library Prep Kit and MiSeq Reagent Kit V3, with sequencing performed at the Finnish Institute for Molecular Medicine, Helsinki. NextSeq 2000 libraries were prepared using the Illumina DNA Prep Kit and IDT 10 bp UDI indices and sequenced at SeqCenter (Pittsburgh, USA). Demultiplexing, quality control, and adapter trimming of Illumina reads were performed with bcl-convert v3.9.3. PacBio Sequel II samples were first fragmented (g-TUBE, Covaris, USA), and libraries were prepared using the SMRTbell Prep Kit v3.0 with barcoded adapter plate v3.0. The Sequel II Binding Kit v3.2 was used for binding and cleanup, and libraries were run on a PacBio Sequel IIe for 15 hours and demultiplexed and trimmed using Lima v2.6.0 (5). Nanopore sample libraries were prepared using ONT Native Barcoding Kit 24 V14 (SQK-NBD114.24) and run on a MinION Mk1B with R10.4.1 flow cells. Demultiplexing was done using Guppy v6.3.8 with the “Super Accurate” base calling model (6).

Trycycler v0.5.3 (7) was used to generate long-read consensus assemblies for each species with Flye v2.9.1-b1780 (HiFi and ONT) (8), Canu v2.2 (HiFi) (9), hifiasm v0.16.1 (HiFi) (10), Miniasm v0.3-r179 (ONT) (11), and Raven v1.8.1 (ONT) (12) following the Trycycler wiki. Long-read assemblies were then polished with short reads using Polypolish v0.5.0 (13) and POLCA from MaSuRCA v4.0.9 (14). Short reads were also independently assembled for each species using Unicycler v0.5.0 (15) with SPAdes v3.15.5 (16) and inspected for circularized plasmids missed by the other assemblers. Assemblies were annotated with the Prokaryotic Genome Annotation Pipeline 2023-10-03.build7061 (17). CheckM v1.2.2 (18) and GTDB-Tk v2.1.1 database 207v2 (19) were used to estimate completeness (all genomes >99% complete) and for taxonomic classification (all genomes classified to species level). All genomes contained only closed circular contigs (cleanly circularized using the reconcile command from Trycycler with default parameters), except *Agrobacterium tumefaciens*, which has one linear chromosome (20). Fourteen species have probable plasmids, as assessed by MOB Recon v3.0.3 (21).

## Data Availability

All sequence data are available under BioProject PRJNA1047486. Computer code, software logs, and intermediate results files used to construct, inspect, and polish the assemblies are available from gitlab (https://gitlab.utu.fi/slhogl/hambiLongRead/) and zenodo (https://doi.org/10.5281/zenodo.10283210).

## References

[1] Estrela S, Sánchez Á, Rebolleda-Gómez M. 2021. Multi-replicated enrichment communities as a model system in microbial ecology. Front Microbiol 12:657467. doi:10.3389/fmicb.2021.657467 33897672 PMC8062719

[2] Grosskopf T, Soyer OS. 2014. Synthetic microbial communities. Curr Opin Microbiol 18:72–77. doi:10.1016/j.mib.2014.02.002 24632350 PMC4005913

[3] Hogle SL, Ruusulehto L, Cairns J, Hultman J, Hiltunen T. 2023. Localized coevolution between microbial predator and prey alters community-wide gene expression and ecosystem function. ISME J 17:514–524. doi:10.1038/s41396-023-01361-9 36658394 PMC10030642

[4] Cairns J, Jokela R, Hultman J, Tamminen M, Virta M, Hiltunen T. 2018. Construction and characterization of synthetic bacterial community for experimental ecology and evolution. Front Genet 9:312. doi:10.3389/fgene.2018.00312 30154827 PMC6102323

[5] Lima:The PacBio barcode demultiplexer and primer remover (v2.6.0). 2022 Pacific Biosciences, USA. Available from: https://lima.how

[6] 2022. Guppy (v6.3.8) with super accurate basecalling (Model: Dna_r10.4.1_e8.2_400bps_modbases_5mc_cg_sup.Cfg). Oxford Nanopore Technologies. Available from: https://nanoporetech.com/support

[7] Wick RR, Judd LM, Cerdeira LT, Hawkey J, Méric G, Vezina B, Wyres KL, Holt KE. 2021. Trycycler: consensus long-read assemblies for bacterial genomes. Genome Biol 22:266. doi:10.1186/s13059-021-02483-z 34521459 PMC8442456

[8] Kolmogorov M, Yuan J, Lin Y, Pevzner PA. 2019. Assembly of long, error-prone reads using repeat graphs. Nat Biotechnol 37:540–546. doi:10.1038/s41587-019-0072-8 30936562

[9] Koren S, Walenz BP, Berlin K, Miller JR, Bergman NH, Phillippy AM. 2017. Canu: scalable and accurate long-read assembly via adaptive K-mer weighting and repeat separation. Genome Res 27:722–736. doi:10.1101/gr.215087.116 28298431 PMC5411767

[10] Cheng H, Concepcion GT, Feng X, Zhang H, Li H. 2021. Haplotype-resolved de novo assembly using phased assembly graphs with hifiasm. Nat Methods 18:170–175. doi:10.1038/s41592-020-01056-5 33526886 PMC7961889

[11] Li H. 2016. Minimap and miniasm: fast mapping and de novo assembly for noisy long sequences. Bioinformatics 32:2103–2110. doi:10.1093/bioinformatics/btw152 27153593 PMC4937194

[12] Vaser R, Šikić M. 2021. Time- and memory-efficient genome assembly with raven. Nat Comput Sci 1:332–336. doi:10.1038/s43588-021-00073-4 38217213

[13] Wick RR, Holt KE. 2022. Polypolish: short-read polishing of long-read bacterial genome assemblies. PLoS Comput Biol 18:e1009802. doi:10.1371/journal.pcbi.1009802 35073327 PMC8812927

[14] Zimin AV, Marçais G, Puiu D, Roberts M, Salzberg SL, Yorke JA. 2013. The MaSuRCA genome assembler. Bioinformatics 29:2669–2677. doi:10.1093/bioinformatics/btt476 23990416 PMC3799473

[15] Wick RR, Judd LM, Gorrie CL, Holt KE. 2017. Unicycler: resolving bacterial genome assemblies from short and long sequencing reads. PLoS Comput Biol 13:e1005595. doi:10.1371/journal.pcbi.1005595 28594827 PMC5481147

[16] Prjibelski A, Antipov D, Meleshko D, Lapidus A, Korobeynikov A. 2020. Using SPAdes De Novo assembler. Curr Protoc Bioinformatics 70:e102. doi:10.1002/cpbi.102 32559359

[17] Tatusova T, DiCuccio M, Badretdin A, Chetvernin V, Nawrocki EP, Zaslavsky L, Lomsadze A, Pruitt KD, Borodovsky M, Ostell J. 2016. NCBI prokaryotic genome annotation pipeline. Nucleic Acids Res 44:6614–6624. doi:10.1093/nar/gkw569 27342282 PMC5001611

[18] Parks DH, Imelfort M, Skennerton CT, Hugenholtz P, Tyson GW. 2015. Checkm: assessing the quality of microbial genomes recovered from isolates, single cells, and metagenomes. Genome Res 25:1043–1055. doi:10.1101/gr.186072.114 25977477 PMC4484387

[19] Chaumeil P-A, Mussig AJ, Hugenholtz P, Parks DH. 2019. GTDB-TK: a toolkit to classify genomes with the genome taxonomy database. Bioinformatics 36:1925–1927. doi:10.1093/bioinformatics/btz848 31730192 PMC7703759

[20] Ren Z, Liao Q, Karaboja X, Barton IS, Schantz EG, Mejia-Santana A, Fuqua C, Wang X. 2022. Conformation and dynamic interactions of the multipartite genome in Agrobacterium tumefaciens. Proc Natl Acad Sci USA 119:e2115854119. doi:10.1073/pnas.2115854119 35101983 PMC8833148

[21] Robertson J, Nash JHE. 2018. MOB-suite: software tools for clustering, reconstruction and typing of plasmids from draft assemblies. Microb Genom 4:e000206. doi:10.1099/mgen.0.000206 30052170 PMC6159552

